# Hemodialysis treatment in patients with severe electrolyte disorders: Management of hyperkalemia and hyponatremia

**DOI:** 10.1111/hdi.12845

**Published:** 2020-05-20

**Authors:** Markus Pirklbauer

**Affiliations:** ^1^ Department of Internal Medicine IV—Nephrology and Hypertension Medical University Innsbruck Innsbruck Austria

**Keywords:** Hemodialysis, hyperkalemia, hyponatremia

## Abstract

Significant deviations of serum potassium and sodium levels are frequently observed in hospitalized patients and are both associated with increased all‐cause and cardiovascular mortality. The presence of acute or chronic renal failure facilitates the pathogenesis and complicates the clinical management. In the absence of reliable outcome data in the context of dialysis prescription, requirement of renal replacement therapy in patients with severe electrolyte disturbances constitutes a therapeutic challenge. Recommendations for intradialytic management are based on pathophysiologic reasoning and clinical observations only, and as such, heterogeneous and limited to expert opinion level. This article reviews current strategies for the management of severe hyperkalemia and hyponatremia in hemodialysis patients.

## POTASSIUM MANAGEMENT

Severe hyperkalemia is defined as serum potassium >6 or >5.5 mEq/l with clinical signs such as arrhythmia or other electrocardiogram (ECG) abnormalities (e.g., T‐wave elevation, loss of P‐wave or sinus‐wave QRS pattern), muscle weakness, and/or ascending paralysis.^1^, ^2^ The causes of hyperkalemia in acute and chronic renal failure include impaired potassium excretion (due to an acute or permanent loss of functioning nephrons, impaired distal tubular flow, hyporeninemic hypoaldosteronism, or medication interfering with potassium excretion in the setting of maintained dietary potassium intake) and altered distribution between the intracellular and extracellular space (e.g., loss of sodium–potassium ATPase activity due to malnutrition, insulin deficiency, or medication like beta‐blocking agents, release after cell breakdown, etc.).

### Hyperkalemia in chronic hemodialysis patients

Observational evidence from a large cohort of incident and prevalent chronic hemodialysis (HD) patients (*n* = 81.013) suggests a U‐shaped relationship between predialysis serum potassium levels and both, all‐cause and cardiovascular mortality. After correction for multiple confounders, these associations remained significant for hyperkalemia. Hypokalemia, however, was no longer associated with all‐cause mortality and only modestly with cardiovascular mortality.^3^ While the pathogenesis of hyperkalemia in end‐stage renal disease (ESRD) is mainly based on diet, medication, and reduced potassium excretion with failing kidney function,^4^ intradialytic management remains a clinical challenge. Intradialytic potassium removal essentially occurs in the first 2 hours of HD treatment and is driven by convective and diffusive transfer. The latter primarily depends on the serum‐to‐dialysate potassium gradient but is also related to intracellular potassium storage capacity and intracellular‐to‐extracellular potassium gradient. During the end of dialysis treatment and during the first hours thereafter, a postdialytic potassium rebound resulting from intracellular‐to‐extracellular translocation occurs. In general, dialysate potassium prescription is based on predialysis serum potassium levels. There is, however, no consensus or randomized controlled trial evidence regarding optimal dialysate potassium choice in chronic HD patients, neither for normal nor abnormal serum potassium levels, resulting in heterogeneous recommendations based on expert opinion level.

In clinical practice, a dialysate potassium of 4 mEq/l is regularly used when predialysis serum potassium levels are ≤4 mEq/l in order to avoid postdialytic hypokalemia (e.g., in malnourished patients) and a dialysate potassium of 3 mEq/l if serum potassium levels range between 4.1 and 5.5 mEq/l. However, a lower dialysate potassium of 2 mEq/L might be used to decrease the risk of hyperkalemia prior to the next HD session in patients with chronic hyperkalemia.^5^ Predialysis serum potassium levels between 5.6 and 8 mEq/l are most commonly treated with dialysate potassium of 2 mEq/l. Persisting hyperkalemia should prompt a review of patient's diet and medication as well as an evaluation of dialysis access recirculation and Kt/V. When serum potassium levels exceed 8 mEq/l, HD might be initiated using a dialysate potassium of 1 mEq/l in order to rapidly decrease potassium to safer levels. Whenever using dialysate potassium of 1 mEq/l, concomitant telemetry monitoring and 30 to 60 minute potassium checks are recommended, and dialysate potassium is usually switched to 2 mEq/l as soon as serum potassium levels decline below 7 mEq/l.^5^, ^6^ A second HD treatment few hours after the first session can be alternatively used to avoid potassium baths <2 mEq/l while still adequately addressing postdialytic potassium rebound (Table 1). Despite most effective in reducing serum potassium levels during HD,^7^ zero dialysate potassium bath is avoided by most dialysis facilities due to the presumed risk of arrhythmia,^8^, ^9^ though, the data supporting this cautious practice are inconsistent.^10^, ^11^ Certain dialysis facilities generally do not lower dialysate potassium bath below 3 mEq/l in patients with high risk for arrhythmia, such as coronary heart disease, left ventricular hypertension, digitalis use, and advanced age.^6^ The latter strategy results from large observational study evidence suggesting an increased risk for sudden cardiac death and arrhythmia with dialysate potassium <2 mEq/l.^12^, ^15^ Based on these findings, several nephrologists suggested to avoid dialysate potassium baths below 2 mEq/l^16^, ^17^ and others even recommend to use decreasing dialysate potassium profiling to maintain a constant intradialytic potassium gradient between dialysate and blood as this might decrease CV risk.^18^, ^19^ In arrhythmia‐prone chronic HD patients, application of such a “constant potassium gradient” approach was associated with lower risk for arrhythmic activity compared to constant low dialysate potassium concentrations.^18^ A large observational analysis from the dialysis outcomes and practice patterns study recently found an equivalent risk for all‐cause death and arrhythmia between dialysate potassium bath of 2 vs. 3 mEq/l and no link between dialysate potassium concentration and next‐session serum potassium levels in maintenance HD patients.^20^ However, as the aforementioned observational studies predominantly included normokalemic maintenance HD patients, these findings might not be representative for patients with chronic severe hyperkalemia in whom low dialysate potassium concentrations are essential to achieve normal range serum potassium levels after the postdialytic potassium rebound.^21^ In this regard, two large observational studies found no association between low dialysate potassium concentrations and the risk of sudden cardiac death in the subgroup of severely hyperkalemic HD patients.^13^, ^14^ Furthermore, low dialysate potassium was associated with fewer sudden cardiac deaths when monthly predialysis serum potassium levels were >5.5 mEq/l.^22^ Finally, no evidence for increased all‐cause mortality even with the use of dialysate potassium baths below 1 mEq/l was found in the hyperkalemic subgroup (serum potassium >5 mEq/l) of maintenance HD patients (*n* = 29.057).^3^ Thus, based on the fact that hyperkalemia was consistently found to be associated with increased all‐cause and cardiovascular mortality, Abuelo recently recommended to use low dialysate potassium baths in patients with chronic severe hyperkalemia instead of avoiding it.^1^, ^21^ In the absence of contradictory evidence, the traditional “rule of seven” was resuggested for determining target dialysate potassium bath. Accordingly, the sum of predialysis serum and dialysate potassium concentration should equal 7 mEq/l, that is, an average of 3.5 mEq/l. For example, a dialysate potassium of 2 mEq/l should be chosen if serum potassium is 5 mEq/l. Whenever serum potassium levels exceed 7 mEq/l, the dialysate potassium bath thus should be zero. The rationale behind is the finding that postdialysis serum potassium levels are usually within 0.5 mEq/l of the initial average of serum and dialysate potassium concentrations.^23^ Chronic HD patients dialyzed by the rule of seven had a similar risk of death compared with controls over 5 years of follow‐up in a study with 1267 HD patients.^24^

**Table 1:** Dialysate potassium prescription in chronic hemodialysis patients

Predialysis serum potassium (mEq/l)	Dialysate potassium (mEq/l)
≤4.0	3 or 4 (based on individual trend)
4.1–5.5	2 or 3 (based on individual trend)
>5.5–8.0	2
>8.0	1 + telemetry monitoring + 30 min K checks and switch to K 2 if serum K < 7
Optional: Prompt consecutive HD to avoid dialysate K < 3 in arrhythmia prone pts.	

HD = hemodialysis; K = potassium.

While the aforementioned studies exclusively evaluated extracellular potassium kinetics, future studies should additionally focus on cellular mechanisms of potassium regulation in HD patients. Altered intracellular potassium storage not only renders some patients more susceptible for developing hyperkalemia (e.g., those with reduced sodium–potassium ATPase activity) but might also explain the heterogeneous study findings regarding dialysate potassium prescription and clinical outcome: Patients' tolerability toward the extent and rate of extracellular potassium removal is mostly related to the ensuing intracellular‐to‐extracellular potassium shift, which is determined by intracellular potassium storage capacity and release mechanisms. Considering individual differences in cellular potassium storage and release, though hard to assess, might be important for a better understanding and management of hyperkalemia in HD patients.

Nondialytic approaches to control hyperkalemia in maintenance HD patients involve the use of gastrointestinal potassium binders that are frequently administered in clinical practice. This therapeutic strategy, however, has not been demonstrated yet to decrease the incidence of predialysis hyperkalemia. In that context, currently ongoing clinical trials will provide further insights in the near future.^25^

### Hyperkalemia in acute HD patients

Acute HD treatment is mandatory whenever acute renal failure presents with severe hyperkalemia due to the impending risk of fatal arrhythmia. Severe hyperkalemia is defined as serum potassium >6 or > 5.5 mEq/l with clinical signs such as arrhythmia or other ECG abnormalities (e.g., T‐wave elevation, loss of P‐wave or sinus‐wave QRS pattern), muscle weakness, and/or ascending paralysis.^1^, ^2^ To immediately antagonize ECG changes, calcium chloride or calcium gluconate is given intravenously over 1 to 2 minutes. Insulin plus glucose, albuterol, and/or sodium bicarbonate can be administered intravenously to transiently shift potassium toward the intracellular space while preparing emergency HD.^5^ The choice of dialysate potassium bath in acute HD is based on predialysis serum potassium levels. In the absence of outcome data, however, available recommendations are based on limited evidence from the maintenance HD population (see above) and clinical observations only. For example, acute renal failure in nonoliguric patients or oliguric patients with low potassium intake frequently presents with normokalemia or even hypokalemia, and thus, a dialysate potassium bath of 4 mEq/l is regularly used in these patients to avoid further potassium lowering. The latter is augmented by acidosis correction and application of total parenteral nutrition.^5^ In nonsevere hyperkalemia (4.5–5.5 mEq/l), a dialysate potassium of 3 mEq/l is commonly used. In patients with ongoing risk of hyperkalemia (e.g., prolonged rhabdomyolysis) and serum potassium >5.5 mEq/l, though, a dialysate potassium bath of 2 mEq/l might be necessary to achieve normokalemia after postdialytic potassium rebound. However, as acute kidney injury and potassium excretion might rapidly recover and low dialysate potassium concentrations (i.e., <3 mEq/l) tend to decrease potassium to or even below normal range, some nephrologists do not lower dialysate potassium <3 mEq/l, especially in arrhythmia‐prone patients. When serum potassium levels exceed 8 mEq/l, rapid potassium removal can be effectively achieved by using a dialysate potassium bath of 1 mEq/l. Whenever using such very low dialysate potassium baths (i.e., <2 mEq/l), ECG telemetry monitoring and frequent potassium checks (every 30–60 minutes) are mandatory and dialysate potassium should be switched to 2 mEq/l as soon as serum potassium levels decline below 7 mEq/l.^6^ A second HD treatment few hours after the first session can be alternatively used to avoid very low dialysate potassium concentrations while still adequately addressing postdialytic potassium rebound (Table 2). Despite conflicting data from the maintenance HD population, zero dialysate potassium bath is generally not recommended in acute HD.^6^ Redistributive potassium‐lowering therapy that transiently shifts potassium toward the intracellular space (i.e., insulin + glucose, albuterol and sodium bicarbonate) should be stopped upon HD initiation in order to increase intradialytic potassium removal and lower postdialytic potassium rebound. In this regard, a glucose‐free dialysate has been demonstrated to increase potassium removal compared to standard dialysate glucose concentrations,^26^ though, this approach has not been implemented in clinical practice yet. Review of diet for high‐potassium containing foods and the use of gastrointestinal potassium binders are suggested for persisting hyperkalemia.^5^

**Table 2:** Dialysate potassium prescription in acute hemodialysis patients

Predialysis serum potassium (mEq/l)	Dialysate potassium (mEq/l)
<4.5	4
4.5–5.5	3
>5.5–8.0	2
>8.0	1 + telemetry monitoring + 30 min K checks and switch to K 2 if serum K < 7
Optional: prompt consecutive HD to avoid dialysate K < 3 in arrhythmia prone pts.	

HD = hemodialysis; K = potassium.

## SODIUM MANAGEMENT

Hyponatremia is a common water balance disorder defined as serum sodium concentration ≤135 mEq/l. Clinical symptoms include nausea, headache, confusion, cognitive deficits, gait disturbances, fatigue, muscle weakness, and cramps but might also be completely absent in mild to moderate hyponatremia (i.e., serum sodium 125–135 mEq/l). Severe hyponatremia (i.e., serum sodium <120 mEq/l) is a potentially life‐threatening disorder with severe neurological complications that can result from cerebral edema or osmotic demyelination in the context of inadequate or excessive treatment, respectively.^27^ Acute hyponatremia is characterized by disease onset <48 hours, whereas chronic hyponatremia gradually develops over several days to weeks. While a discussion of the multifactorial etiology of hyponatremia in ESRD is far beyond the scope of the present review, there is a large body of evidence demonstrating that chronic hyponatremia is more common (6%–29%), related to malnutrition and loss of residual kidney function, and independently associated with mortality in prevalent and incident HD patients.^28^, ^29^ The causal relationship between hyponatremia and mortality still remains to be elucidated but might involve, among others, central nervous toxicity, increased falls and fracture risk, infection‐related complications as well as impaired cardiac function.^29^ Hyponatremia is also frequently observed and associated with poor prognosis in patient with acute renal failure, especially in critically ill with multiple predisposing conditions, such as impaired free water excretion, frequent administration of hypotonic fluids, and polypharmacy.^30^

### Normonatremia in chronic HD patients

Recommendations for dialysate sodium prescription in chronic HD patients with normal serum sodium levels are based on pathophysiologic considerations, limited observational data, and expert opinion only. Average prescribed dialysate sodium concentrations increased from 130 mEq/l in the 1960s to 138–141 mEq/l in 2000. Since 2010, a significant increase in the range of prescribed dialysate sodium concentrations (between 136 and 149 mEq/l) could be observed among maintenance HD patients, reflecting the lack of outcome data and heterogeneous clinical practice.^31^ While some dialysis facilities use fixed dialysate sodium concentrations independent of patients' serum sodium levels in the chronic HD setting, other facilities individualize dialysate sodium prescription based on predialysis serum sodium levels, blood pressure and ultrafiltration tolerability. While neither of these two strategies has proven superiority, it has been previously demonstrated that next‐session serum sodium levels are generally not associated with dialysate sodium concentrations, independent of the facility's dialysate sodium prescription practice (fixed vs. individualized).^28^, ^32^ However, dialysate sodium >138 mEq/l has been previously associated with increased thirst and intradialytic weight gain and concentrations <136 mEq/l might favor intradialytic hypotension and cramps. A large observational study suggested a moderate survival benefit with the use of higher dialysate sodium concentrations among hyponatremic patients.^28^

### Normonatremia in acute HD patients

There is no general recommendation for dialysate sodium prescription in normonatremic patients. From a pathophysiologic perspective, dialysate sodium concentrations above serum sodium levels increase postdialysis serum sodium and lead to an increase in sympathetic tone, thirst, intradialytic weight gain, volume expansion, and hypertension. A dialysate sodium concentration of more than 2 to 3 mEq/l below serum sodium levels decreases postdialysis sodium and osmolality^33^ and can negatively affect ultrafiltration (UF) tolerance. The latter could enhance intradialytic hypotension, myocardial ischemia, and thus cardiovascular mortality.^31^

In clinical practice, dialysate sodium prescription ranges from 136 to 145 mEq/l and is based on predialysis sodium levels as well as hemodynamic status. Basically, dialysate sodium concentrations of up to 3 mEq/l below serum levels should result in zero diffusive sodium transfer; however, differences between prescribed and measured inlet dialysate sodium have to be considered.^34^

### Severe acute hyponatremia in acute and chronic HD patients

In patients with severe acute hyponatremia, that is, hyponatremia developing <48 hours (e.g., during water poisoning), and concomitant need for acute or chronic renal replacement therapy, aggressive correction of serum sodium levels as in the nondialysis population is mandatory due to the risk of cerebral edema.^35^ Symptomatic treatment with hypertonic saline (3%) is possible but should be avoided in hypervolemic patients. Vasopressin (V2) receptor antagonists are not recommended. Conventional HD with standard dialysate sodium concentrations is the treatment of choice in acute and chronic HD patients^6^ (Table 3).

**Table 3:** Management of severe acute and chronic hyponatremia in hemodialysis patients

Severe acute hyponatremia Na < 120 mEq/l Onset < 48 h	3% Saline bolus (150 ml i.v.) for severe symptoms (avoid in hypervolemic patients)
	Rapid correction using intermittent HD (dialysate Na 136–145 mEq/l)
	Vasopressin receptor antagonists *not* recommended
Severe chronic hyponatremia Na < 120 mEq/l Onset > 48 h	3% Saline bolus (150 ml i.v.) for severe symptoms (avoid in hypervolemic patients)
	Daily short HD with lowest dialysate Na (= 130 mEq/l) and low blood flow (50–100 ml/h) *or* daily CVVH with customized replacement fluid Na concentration (Na kinetic modeling)
	Recommended serum Na correction rate is 4–8 mEq/l per 24 h
	Hourly serum Na checks, 5% dextrose in water (i.v.) if correction rate exceeded
	Vasopressin receptor antagonists *not* recommended

CVVH = continuous venovenous hemofiltration; HD = hemodialysis; Na = sodium.

### Severe chronic hyponatremia in acute and chronic HD patients

HD patients presenting with severe chronic hyponatremia (i.e., serum sodium <120 mEq/l) represent a therapeutic challenge both in the acute and chronic HD settings. In the presence of chronic hyponatremia gradually developing over days to weeks, adaptation of brain cells reduces the risk of cerebral edema by excreting organic osmolytes.^36^ This adaptation, however, renders brain cells vulnerable to osmotic demyelination syndrome (i.e., pontine and extrapontine myelinolysis) in the event of rapid serum sodium correction, which, thus, has to be essentially avoided.^37^, ^38^, ^39^, ^40^, ^41^ Uremia might have some protective potential against osmotic demyelination but there are case reports demonstrating osmotic demyelination syndrome after HD treatment in severely hyponatremic ESRD patients.^42^, ^43^ Hence, latest recommended correction rate for the treatment of severe chronic hyponatremia in acute and chronic HD patients is 4 to 8 mEq/l per 24 hours as in the non‐ESRD population.^27^ Symptomatic treatment of hyponatremia with hypertonic saline (3%) is possible but usually avoided in patients with kidney failure and volume expansion. Vasopressin (V2) receptor antagonists are not effective at reduced glomerular filtration rates and therefore not recommended in ESRD.^44^ As the lowest available dialysate sodium concentration in intermittent HD is 130 mEq/l, it remains a therapeutic challenge to achieve low intradialytic sodium correction rates in patients with severe chronic hyponatremia. A potential approach is lowering of dialysis blood flow to slow intradialytic sodium transfer to the blood: The total amount of sodium required to achieve a desired serum sodium increase in 24 hours depends on total body water (i.e., desired serum sodium increase in 24 hours × total body water). The calculated total sodium amount divided by sodium transfer rate, which is determined by the dialysate‐to‐blood sodium gradient, allows to calculate the required total blood flow for targeted sodium transfer. By reducing HD session length to 120 to 180 minutes, the resulting blood flow rate will be in a low (e.g., 50–100 ml/minute), but still practicable range. Thus, conventional HD with low blood flow, lowest available dialysate sodium concentration, that is, 130 mEq/l, and reduced treatment time is a simple strategy to safely correct hyponatremia in the absence of continuous renal replacement therapy options^45^ both in acute and chronic renal failures. Hourly serum sodium checks during the HD session are mandatory and intravenous application of 5% dextrose in water might be required to stay within the target sodium correction rate. Daily application of low blood flow dialysis will help to slowly correct hyponatremia over several days and to compensate for reduced single session Kt/V.^6^ Alternating isolated ultrafiltration might be necessary for achieving volume control in hypervolemic patients.^5^

Utilization of continuous renal replacement therapies, such as continuous venovenous hemofiltration (CVVH), is an alternative treatment option allowing slow sodium correction over a longer period of time. The commercially available replacement fluid (RF) sodium concentration is usually fixed at 140 mEq/l; however, customization of RF by adding or replacing water allows to achieve slow sodium correction rates with CVVH.^46^ Calculating the required RF sodium necessitates the use of sodium kinetic models. While previous complex modeling approaches already demonstrated their accuracy to predict end of treatment sodium levels,^47^ much simpler kinetic equations for customization of RF sodium concentration, that are readily applicable in clinical practice, have been published by Yessayan et al.^48^:

(1) $$\text{Replacement fluid}\;\mathrm{Na}=\text{initial serum}\;\mathrm{Na}+\frac{\text{desired}\;\Delta \;\text{serum}\;\mathrm{Na}}{\left(1-e^{\frac{\left(-D\times 24\right)}{V}}\right)}.$$

Based on initial serum sodium concentration, desired change (Δ) in serum sodium concentration, total body water (*V*), and sodium dialysance (D), kinetic modeling allows to estimate sodium concentration in the RF that is required to achieve a desired serum sodium change after 24 hours of treatment (Equation 1). Recalculation of RF sodium concentration based on changes in total body weight and serum sodium levels is mandatory at least every 24 hours.

Depending on CVVH mode (predilution vs. postdilution), sodium dialysance can be estimated as follows:

$$D\;\left(\text{predilution}\right)=\left(\frac{\mathrm{Qb}}{\mathrm{Qb}+\mathrm{Qrf}}\right)\times \text{SCNa}\times \left(\mathrm{Qrf}+\mathrm{Quf}\right),$$

(2) $$D\;\left(\text{postdilution}\right)=\text{SCNa}\times \left(\mathrm{Qrf}+\mathrm{Quf}\right),$$

where Qb = blood flow rate (l/hour), Qrf = RF flow rate (l/hour), Quf = ultrafiltration rate (l/hour), SCNa = Na sieving coefficient (~1).

To reduce RF sodium concentrations in a graded manner every 24 hours, successive dilutions of RF bags can be made by adding sterile water to standard RF bags. The required volume can be calculated as follows:

$$\text{Volume to}\;\mathrm{add}=\frac{\mathrm{RF}\;\text{volume}\times \left(\text{initial}\;\mathrm{RF}\;\mathrm{Na}-\text{desired}\;\mathrm{RF}\;\mathrm{Na}\right)}{\text{initial}\;\mathrm{RF}\;\mathrm{Na}},$$

(3) $$\text{Volume to exchange}=\mathrm{RF}\;\text{volume}-\frac{\text{desired}\;\mathrm{RF}\;\mathrm{Na}\times \mathrm{RF}\;\text{volume}}{\text{initial}\;\mathrm{RF}\;\mathrm{Na}}.$$

Using customized RF solutions with CVVH is considered a safe way to achieve low sodium correction rates. Application of these kinetic equations has already proven its efficacy in clinical practice and allows to gradually increase serum sodium levels in patients with severe hyponatremia and concomitant need for acute and chronic renal replacement therapy. Recustomization of the RF sodium concentration every 24 hours is mandatory^48^ (Table 3). Paquette et al. similarly demonstrated that the aforementioned kinetic equations can be used in clinical practice to slowly correct severe chronic hypernatremia during CVVH by customizing RF sodium via the addition of sodium chloride to the RF bag.^49^ This is of clinical importance as intermittent HD is not recommended in severely hypernatremic patients.

## CONCLUSIONS

Optimal choice of dialysate potassium and sodium concentration still remains a clinical challenge both in patients with normal and abnormal electrolyte levels. The lack of outcome data has led to heterogeneous recommendations based on pathophysiologic considerations and clinical practice only. This article reviews current strategies for potassium and sodium management in acute and chronic HD patients. Low dialysate potassium concentrations have recently been considered rather safe in hyperkalemic maintenance HD patients. The use of either intermittent HD with low calculated blood flow and lowest achievable dialysate sodium (i.e., 130 mEq/l) or CVVH with gradually customized RF sodium concentration based on practicable kinetic equations may help to safely manage severe chronic hyponatremia in acute and chronic HD patients.
